# Polyphenylsilsesquioxanes. New structures–new properties

**DOI:** 10.1039/d0ra07854a

**Published:** 2020-11-26

**Authors:** Maxim N. Temnikov, Aziz M. Muzafarov

**Affiliations:** Nesmeyanov Institute of Organoelement Compounds, Russian Academy of Sciences ul. Vavilova 28 Moscow 119991 Russia aziz@ineos.ac.ru; Enikolopov Institute of Synthetic Polymeric Materials, Russian Academy of Sciences ul. Profsoyuznaya 70 Moscow 117393 Russia

## Abstract

The review describes the synthesis and properties of various forms of polyphenylsilsesquioxane (PPSQ). Among the forms described, we present the well-known ladder (l-PPSQ) and polyhedral (p-PPSQ) forms, from the first studies to the latest achievements. The practical prospects of these compounds and the possibility of their modification are estimated. These PPSQ have a regular polycyclic structure, which allowed us to compare them with random polycyclic analogs (r-PPSQ). The last part of the review describes the acyclic PPSQ (a-PPSQ) obtained recently. The methods for their synthesis and modification are presented. Modification of (a-PPSQ) allows two new forms of PPSQ to be obtained. The first one is a hyperbranched PPSQ. The second one is a globular PPSQ or a nanogel as it is called by the authors. Both forms are of great interest because their physicochemical properties differ greatly from the known ones (l-PPSQ, p-PPSQ, r-PPSQ). The areas of practical application of the new PPSQ forms are predicted.

## Abstract 2

The review describes the synthesis and properties of various forms of polyphenylsilsesquioxane (PPSQ).

## Graphical abstract

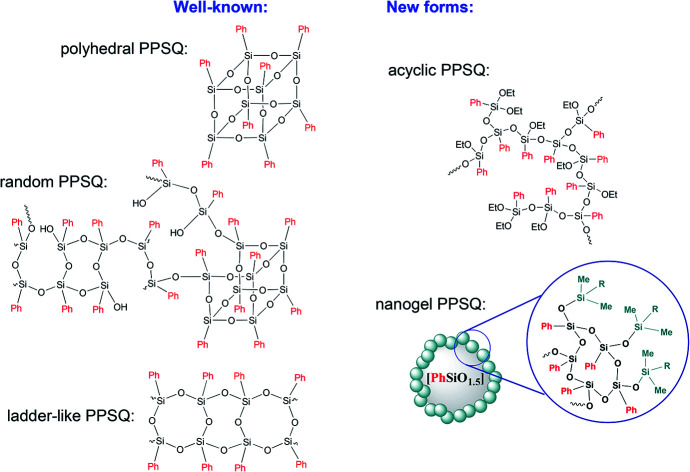

## 1. Introduction

Polyorganosilsesquioxanes with the general formula RSiO_1.5_ represent an important class of organosilicon polymers. Due to the synergy of the properties of organic and inorganic compounds, they are widely used in various fields of industry and in everyday life.^1,2^

A special place in this class of polymers belongs to polyphenylsilsesquioxanes (PPSQ). The phenyl radical at the silicon atom gives these compounds enhanced thermal and radiation stability.^3^ In combination with excellent dielectric properties, hydrophobicity and solubility, this allows them to be widely used as protective coatings for electronic devices.^4,5^ Coatings based on commercial varnishes based on phenylsilsesquioxane (or its copolymers with difunctional monomers) ensure long-term operation of devices under high humidity conditions at 250–300 °C.^6^

The optical transparency and high refractive index make polyphenylsilsesquioxanes attractive objects for optics and optoelectronics. They are used as waveguides^7–10^ and coatings for optical devices.^11–14^

Incorporation of various lanthanide complexes^15–17^ into polyphenylsilsesquioxanes allows one to obtain plastic luminescent materials. The addition of luminophores to PPSQ by chemical reactions appears even more attractive.^18^ Such materials are promising for the formation of multiband luminescent optoelectronic devices.

Incorporation of PPSQ into copolymers improves various physical properties of the latter. For example, its copolymer with polyurethane has enhanced electrical insulating properties,^19^ while incorporation of 25% of polyphenylsilsesquioxane blocks into polydimethylsiloxane (PDMS) noticeably increases the radiation stability of the latter.^20^

The concept of PPSQ incorporation into PDMS has been implemented commercially. In Russia, FGUP NIISK (St. Petersburg) produces a block copolymer under “Lestosil” trademark. In this block copolymer, the rigid PPSQ block combined with flexible polydimethylsiloxane imparts superior mechanical properties to these copolymers. Heat-resistant insulating coatings which combine strength and hardness with high elasticity are obtained on this basis.^21,22^ A similar formulation with an original structure, an analogue of Lestosil–Blocksil, was developed at INEOS RAS (Institute of Organoelement Compounds, Russian Academy of Sciences).^23^

As one can see from the above, PPSQ are used in various human activities. They are used both in pure or copolymer form (varnishes, compounds) and in composite materials. Moreover, PPSQ can be used as a polymer matrix, filler or modifier in composites.

Such a variety of application areas is not due to a special versatility of PPSQ but to the variety of structural forms corresponding to the same chemical composition of the polymer. In the majority of the above cases, the properties of PPSQ for a specific practical application were optimized by empirical selection of synthesis conditions. The sensitivity of the PPSQ structure to the conditions of its synthesis implies that the properties can be optimized in structure–properties coordinates.

**Fig. 1:**
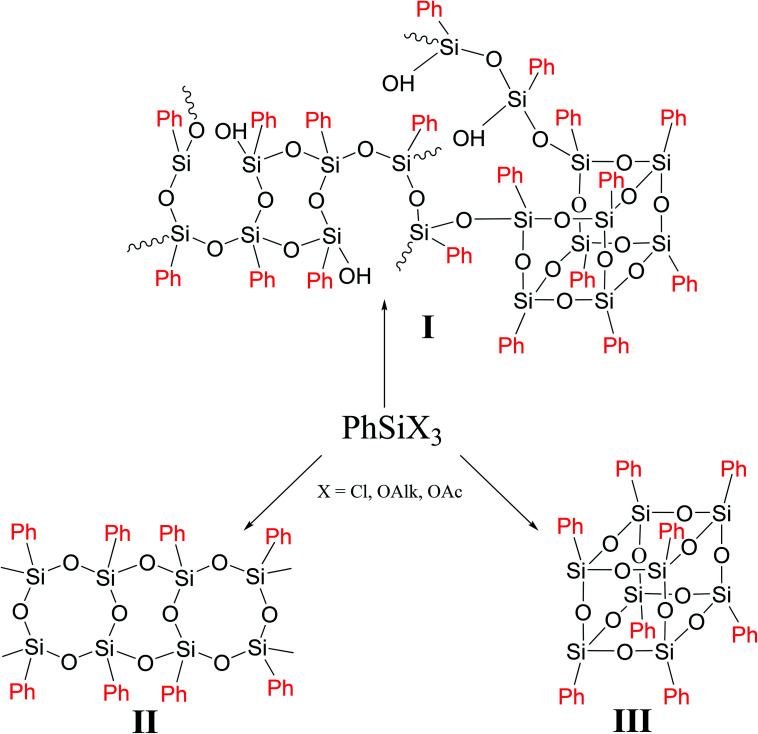
Organosilsesquioxanes with different macromolecular structures.

In general case, PPSQ are prepared by hydrolytic polycondensation of a trifunctional monomer PhSiX_3_, where X = Cl, OAlk or OAc. The main difference between PPSQ and products obtained by polycondensation of other trifunctional organosilanes (for example, methylsilsesquioxanes) is that, along with mixed, so-called cyclolinear polymers (Fig. 1 (I)), compounds with regular structures can also be synthesized (Fig. 1 (II and III)).^2^ In other words, the statistical structure of I completely degenerates into II or III under certain conditions. The ability to form such structures is promising for the development of selective synthetic methods, as well in studies of structure–properties relationships.

As one can see from Fig. 1, though the PPSQ forms vary, they have a common feature. All of them are polycyclic. Methods for the preparation of an acyclic PPSQ were developed rather recently.^24,25^ Obviously, the new PPSQ form may expand its application scope even further. Moreover, we will be able to find out how the new form will affect the properties of the final polymer.

The purpose of this review is to compare the known polycyclic forms of polyphenylsilsesquioxane with the new acyclic form in terms of synthesis and properties.

We will consider regular (ladder, macrocyclic, polyhedral PPSQ) and PPSQ with statistical composition. These compounds will be compared with hyperbranched a-PPSQ and their derivative nanogel form, n-PPSQ. The applied and fundamental potential of the new PPSQ form will be estimated.

## 2. Polycyclic forms of PPSQ

### 2.1. Ladder PPSQ

One of the best known PPSQ representatives has a ladder polymer structure (l-PPSQ) (Fig. 1 (II)). This unique compound has been the focus of studies in many scientific centers for several decades. An l-PPSQ was first synthesized by Brown in 1960.^26^ The approach he used for this involved three stages.^2^ At the first stage, the hydrolysis of phenyltrichlorosilane (PTCS) is performed in a solvent containing excess water. The reaction products have a low molecular weight of about 10^3^. At the second stage, the resulting hydrolyzate is subjected to an equilibrium rearrangement in the presence of a strong base such as KOH. The molecular weight of the so-called prepolymer obtained at this stage is 10^4^. At the final stage, a concentrated solution of the prepolymer is subjected to high-temperature (250 °C) polymerization in the presence of KOH as a catalyst.

The reaction product is soluble in a number of organic solvents and has a molecular weight of 10^5^ to 10^6^. In this case, the glass transition temperature is higher than the decomposition temperature, which indicates that the rigidity of the main macromolecule chain is extremely high.

Due to the ladder structure, l-PPSQ compounds possess a number of unique properties, namely, high thermal and thermooxidative stability (up to 500 °C) and ability to form strong films.^27^ The high molecular weight and good solubility combined with the beneficial properties inherent in PPSQ mentioned above make them very attractive polymeric matrices for a wide range of composite materials.^19,28–32^

**Fig. 2:**
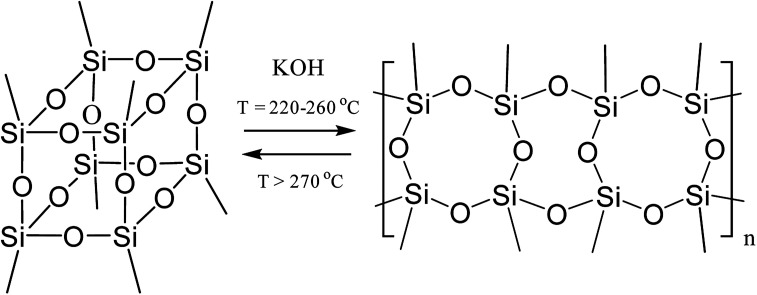
Equilibrium in a ladder/cage-like polymer system.^33^

The formation of such a regular polymer under the drastic conditions of the synthesis indicates the thermodynamic preferability of the polycyclic structure that is formed in this process. It was shown^33^ for the polymerization of a mixture of tolylsilsequioxanes (close analogues of PPSQ) as an example that at temperatures above 270 °C, the ladder/cage-like polymer equilibrium shifted towards the formation of low molecular weight cage-like structures, Fig. 2:

The authors attribute this to entropy factors. In other words, a cage-like structure will be thermodynamically preferable at high temperatures.

The drastic conditions used in the preparation of l-PPSQ result in a certain defectiveness of the polymer structure. This undoubtedly reduces its regularity; negatively, or rather unpredictably, affects the properties, and makes it much more difficult to control them.^34^ Analyzing these defects is not an easy task. The problem of overcoming these drawbacks, as well as the recent high interest in studying the properties of regular polymers, served as a driving force for the search for new selective approaches to the synthesis of highly regular l-PPSQ.

At the first stage, the effect of the pre-organization of precursors was estimated. Switching to disiloxanes and other phenyltrichlorosilane partial hydrolysis products obtained using the ingenious method of vapor-phase hydrolysis developed at INEOS RAS showed that monomers with different numbers of silicon atoms give rise to macromolecules with different hydrodynamic characteristics. This was attributed to differences in the structure of the products.^35^ As a quintessence of this approach, tetraphenylcyclotetrasiloxane-tetraol, or “tetrol”, was used as the starting reagent as a mixture of stereoisomers^36^ or as individual stereoisomers.^37,38^ In this case, the rigidity of ladder macromolecules increased greatly, as it was quantitatively confirmed later.^39^

**Fig. 3:**
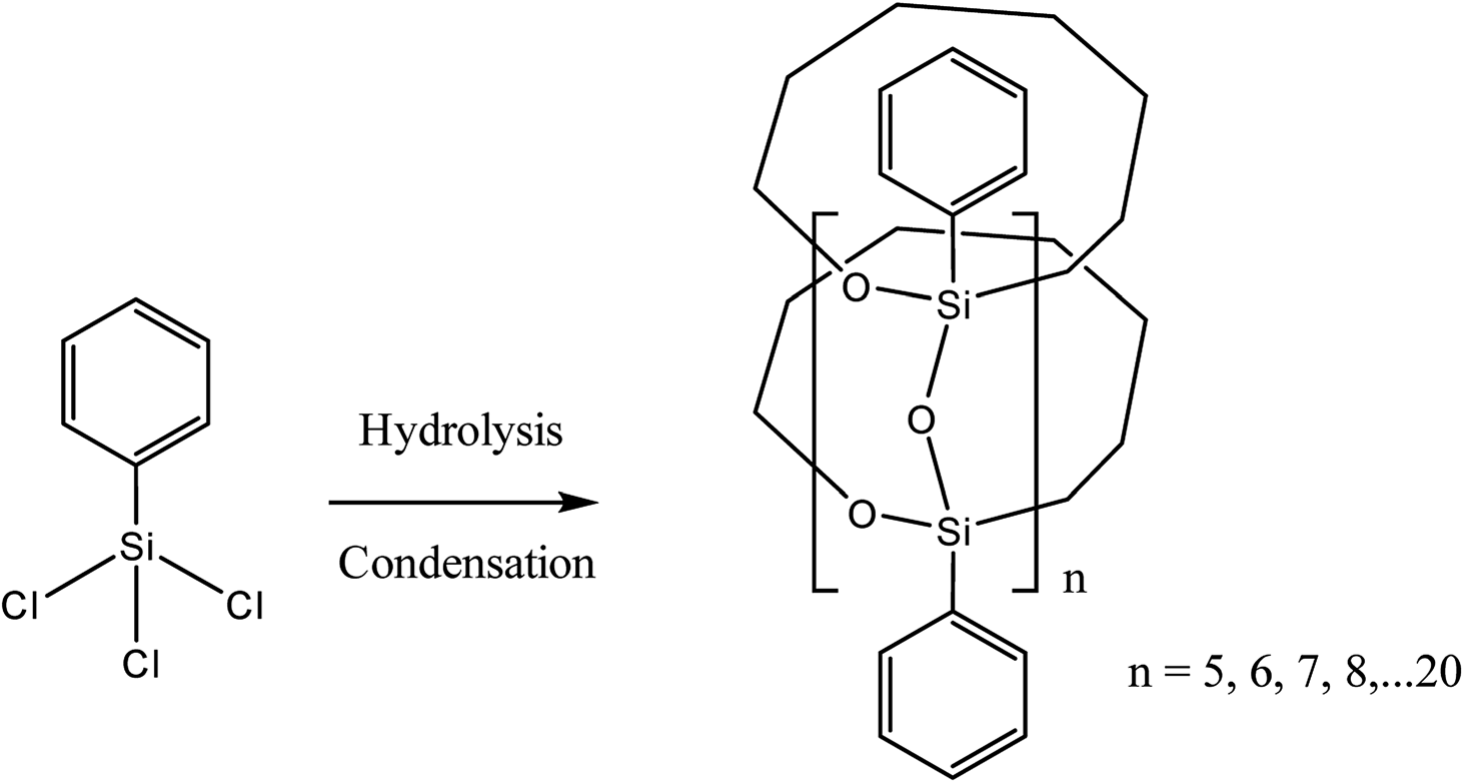
Scheme for the synthesis of a cyclic l-PPSQ. Reprinted with permission from ref. 40. Copyright 2020 American Chemical Society.

It should be noted that the most satisfactory results were achieved by preliminary hydrolysis and/or condensation of organosilicon monomers. The resulting prepolymer was then subjected to anionic polymerization. The structure of such a polymer is also of interest. One of the modern examples describes a method for the preparation of a macrocyclic ladder oligomer^40^ (Fig. 3):

The polymer structure was confirmed by a set of state-of-the-art methods. The methods included PXRD, MALDI-TOF MS, FT-IR, GPC, solid-state ^29^Si NMR, as well as the more conventional ^1^H and ^13^C NMR in CDCl_3_ solution. The data obtained were correlated with computer calculations. It is likely that in the studies cited above (1970–1990), similar (possibly more defective) structures were obtained. However, the level of analytical methods available at that time did not allow the exact structure of the condensation products to be determined.

**Fig. 4:**
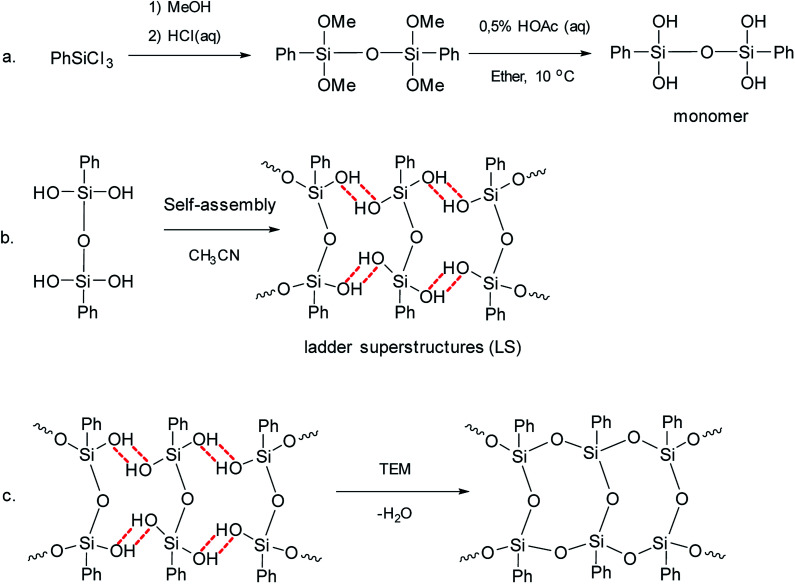
Scheme for the synthesis of l-PPSQ. Reprinted with permission from ref. 41. Copyright 2020 American Chemical Society.

Later, pre-organized predecessors were resorted to again at a new round.^41^ The idea was to organize structural units preliminarily using the hydrogen bonds of the SiOH groups and the π–π stacking of the phenyl substituents. Further, the hydrogen bonds were converted to covalent bonds by a chemical reaction. Thus, the pre-organized (ladder) structure was fixed. This method developed by Zhang includes three sequential steps:

(1) Synthesis of a four-functional monomer (“a” in Fig. 4).

(2) Self-organization of the monomer followed by lyophilization (“b” in Fig. 4). It results in ladder superstructures (LS).

(3) *In situ* polycondensation on an inert flask surface (“c” in Fig. 4).

**Fig. 5:**
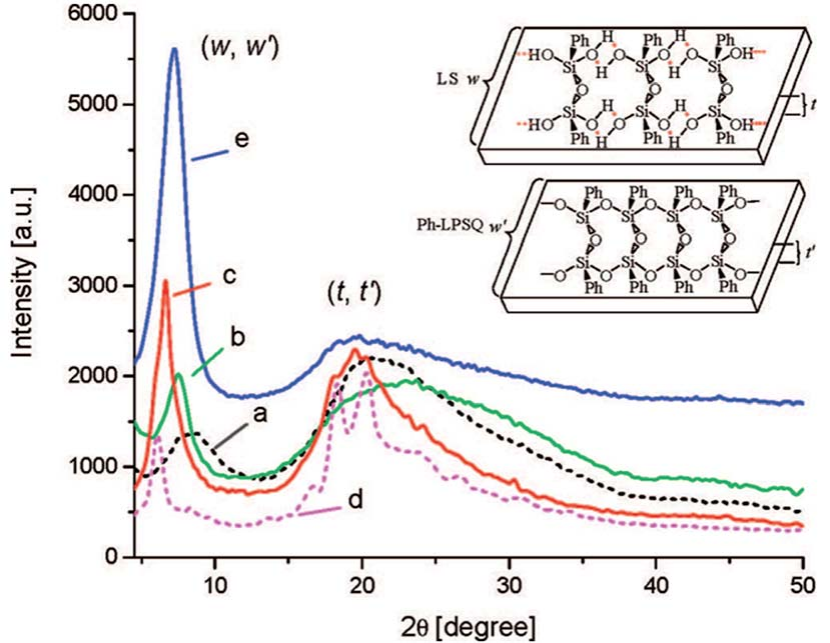
XRD patterns of ladder superstructures (LS) lyophilized from monomer solutions (0.1 mol L^−1^, 0 °C) in different solvents: (a) 1,4-dioxane, (b) acetonitrile, (c) acetonitrile/toluene (1 : 1, v/v), (d) acetonitrile/toluene (1 : 1, v/v) + urea (excess), respectively; (e) XRD pattern of the ladder polymer l-PPSQ prepared *via* monomer self-organization-lyophilization-surface-confined polycondensation. The inserted chart illustrates the ladder width (*w*, *w*′) and ladder thickness (*t*, *t*′) of the ladder superstructure and l-PPSQ, respectively. Reprinted with permission from ref. 41. Copyright 2020 American Chemical Society.

Powder X-ray diffraction (PXRD) data are the main evidence of the ladder structure. Fig. 5 shows the diffractograms for the products obtained:^41^

It can be seen from the figure that if different solvents are used at the stage of LS pre-organization, the width and intensity of the peaks in the diffractogram vary (Fig. 5a–c). It has been shown in previous studies^42,43^ that the peak around 7° corresponds to the intramolecular interchain distance (in other words, the chain width, see Fig. 9, *w*), while that at about 20° corresponds to the chain thickness (see Fig. 9, *t*). It was shown^41^ that these distances well correlate with the results of theoretical calculations. Further evidence about the pre-organized ladder structure in LS is provided by the authors' experiment with addition of urea. Once it was added, the width, maximum and intensity of peaks in the diffractogram (Fig. 5d) changed significantly. This observation was attributed to a violation of the ladder structure due to destruction and rearrangement of hydrogen bonds.

**Fig. 6:**
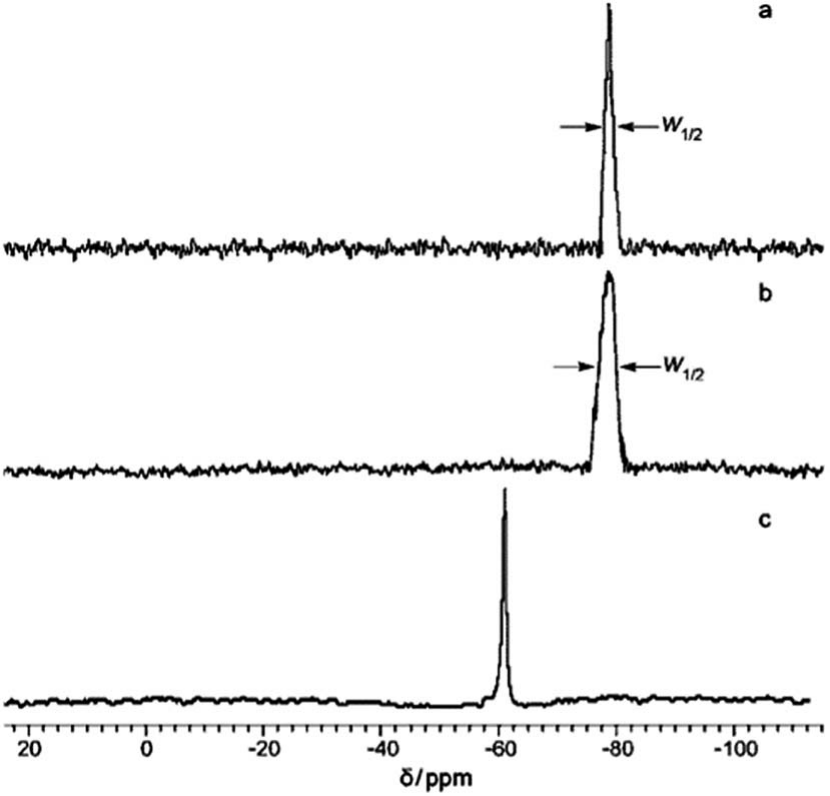
^29^Si NMR spectra of (a) l-PPSQ prepared *via* monomer self-organization-lyophilization-surface-confined polycondensation, (b) l-PPSQ prepared *via* solution polycondensation in acetonitrile, (c) ladder superstructure self-organized in acetonitrile solution at 25 °C. Reprinted with permission from ref. 41. Copyright 2020 American Chemical Society.

In addition to XRD, ^29^Si NMR spectroscopy data can also provide evidence of the ladder structure. Fig. 6 presents the NMR spectra of the products obtained:^41^

One can see from the figure that l-PPSQ has a narrow peak with a chemical shift of about 78–80 ppm (Fig. 6a). If the reaction is performed in acetonitrile, peak broadening is observed (Fig. 6b). In the case of a pre-organized ladder structure, a singlet that can be attributed to the (Ph_2_Si(OH)_2_)_2_O monomer is observed (Fig. 6c). In general, it should be noted that XRD and ^29^Si NMR spectroscopy alone are insufficient to prove a ladder structure. The use of these methods is based on a number of previous studies where the evidence base was much more comprehensive.

If this approach is followed, the resulting l-PPSQ has a molecular weight of up to 2 × 10^4^ (according to GPC) and a glass transition temperature of *T*_g_ = 140–160 °C. The fact that the l-PPSQ synthesized in this work have a *T*_g_ indicates that the actual MW is considerably smaller than that measured by GPC. This can be explained by the conformation of the ladder structure. This conformation is rod-shaped due to an extremely high rigidity of the macromolecular chain. The MW of the l-PPSQ obtained were estimated by GPC using polystyrene standards. In this case, the same hydrodynamic radius of a polystyrene coil and l-PPSQ will correspond to different MW values. The MW of a polystyrene coil is greater than that of l-PPSQ. Therefore, analysis of l-PPSQ based on polystyrene standards should give overestimated MW values.

Though this approach was rather unsuccessful (high molecular weight structures were never obtained), its fundamental difference was that the preliminary self-organization of functional structural elements was attempted. It should be reminded that pre-organized precursors were used in the previously known variants, and their subsequent treatment was performed by the traditional scheme with preliminary precursor condensation followed by anionic polymerization.

**Fig. 7:**
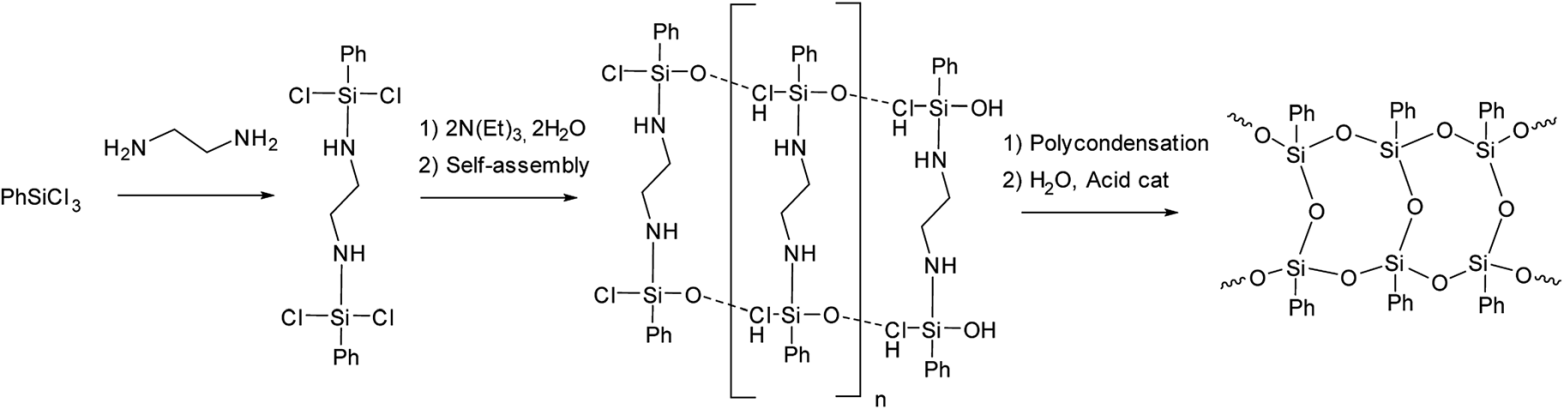
Scheme of synthesizing l-PPSQ by the *endo*-template method.^44^ Reproduced with the permission of Springer.

The idea of preliminary self-organization of structural elements and abandoning polymerization processes was further developed by X. Yang *et al.*^44,45^ who used an *endo*-template approach. It involves the use of ethylenediamine at the stage of phenyltrichlorosilane hydrolysis as shown in Fig. 7:

The l-PPSQ thus obtained had a highly regular structure and a molecular weight of about 5 × 10^4^. Their structure was confirmed by XRD and ^29^Si NMR spectroscopy, like in the previous paper.

It should be noted that this approach was already demonstrated previously elsewhere.^46^ Unlike in this study, *para*-phenylenediamine was used. Moreover, the resulting siloxanesilazane polymer was not converted to l-PPSQ.

**Fig. 8:**
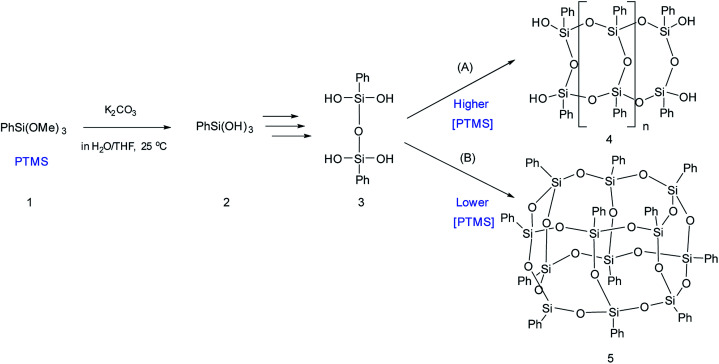
Scheme for synthesizing (A) l-PPSQ and (B) [PhSiO_1.5_]_12_. Reprinted with permission from ref. 47. Copyright 2020 American Chemical Society.

The simplest way for synthesizing ladder l-PPSQ was suggested by S. Choi *et al.*^47^ The hydrolysis of phenyltrimethoxysilane (PTMS) occurred at room temperature in the presence of K2CO3 as a weak base. The molecular weight of the resulting polymer was 1.5 × 10^4^. At the same time, the product contained nearly no residual hydroxy groups. Yet another important conclusion made in the article concerns the concentration of the starting PTMS. In fact, if the initial monomer content was high (above 4.5 M), selective formation of l-PPSQ occurred, whereas polyhedral oligophenylsilsesquioxane (p-PPSQ) with general formula [PhSiO1.5]12 (Fig. 8) was obtained at concentrations below 4.5 M.

It was shown by ^29^Si NMR spectroscopy that, like in the case described above, the formation of a ladder or polyhedral phenylsilsesquioxane was preceded by 1,3-diphenyltetrahydroxydisiloxane (Fig. 8 “3”). It is possible that this monomer subsequently undergoes self-organization. In this case, the concentration determines the pathway of the product formation. K_2_CO_3_ is required for catalyzing the condensation of SiOH groups. It is a weak base that cannot rearrange the siloxane frame under mild conditions.

These works demonstrate the complexity of synthesizing regular l-PPSQ with low defectiveness. On the other hand, decreasing the defectiveness by using milder synthesis conditions results in a decrease in the product's MW. For comparison, we can refer to a work by Unno^48–50^ who developed a complicated but very efficient scheme for synthesizing a ladder polysiloxane with isobutyl substituents at silicon atoms. This unique model work made it possible to synthesize rather a large completely defect-free “ladder”. Unfortunately, the chemical technique used employs phenyl substituents at the silicon atom as the functional groups, and therefore the compounds obtained cannot be used as actual regular models for l-PPSQ. Nevertheless, the study of their properties gave ample data for evaluating the regularity values of this class of polymers.

Despite all the rich prerequisites, PPSQ did not find wide practical application in its original form. However, they aroused great interest as components of copolymer systems (Lestosils and Blocksils, see above) and as objects for chemical modification. For example, incorporation of nitro groups to a phenyl radical by electrophilic substitution improves the compatibility of l-PPSQ with a polyimide matrix.^30^ Subsequent reduction of the nitro group to an amino group resulted in chemical crosslinking of the ladder polymer with the polyamido acid. The composite thus obtained had higher glass transition temperature and Young's modulus. Yet another use of electrophilic substitution for l-PPSQ modification involves the incorporation of sulfonic acid groups at an aromatic substituent.^51^ Conductive heat-resistant films based on modified l-PPSQ and polyaniline were successfully obtained using this approach. The chemical unambiguity of this modification raises many questions due to the possible cleavage of the silicon–phenyl bond that readily occurs under electrophilic conditions.^52^ Apparently, the authors were primarily interested in the properties rather than in the process chemistry.

The examples presented above allow us to conclude that l-PPSQ that were first obtained in the 60 s of the past century are still of interest for scientists. This is due to their unique structure that makes them promising objects for further studies on the structure–properties relationships. The specifics of the synthesis make it possible to vary the molecular weight and regularity of the resulting polymers, which noticeably affects the properties of the latter. Moreover, modifying l-PPSQ allows one to expand its application field considerably. This is particularly promising, taking into consideration the potential of new methods for PPSQ synthesis under mild conditions.

### 2.2. Polyhedral polyphenylsilsesquioxanes

Polyhedral polysilsesquioxanes (p-PPSQ) (Fig. 1 (III)) represent yet another well studied class of compounds. They attract particular attention as building blocks for nanocomposite materials,^53–59^*i.e.*, objects used in catalytic systems^60,61^ and as precursors for the preparation of porous media.^62^

**Fig. 9:**
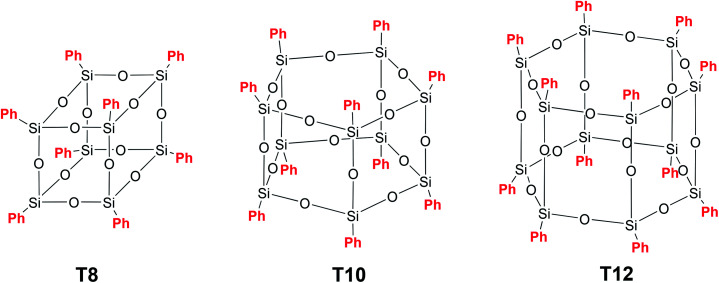
Structures of the most common polyhedral PPSQ.

At the same time, the most studied representatives of this class of phenylsilsesquioxanes, namely, [PhSiO1.5]_8_ (T_8_), [PhSiO1.5]10 (T_10_) and [PhSiO1.5]12 (T_12_) (Fig. 9), do not attract particular attention, mainly due to poor solubility in the majority of organic solvents.^63^

However, as it will be shown below, when they are modified by electrophilic substitution at the phenyl ring, it is possible to improve the solubility considerably and, accordingly, expand the application scope of such compounds. The synthesis of polyhedral phenylsilsesquioxanes is possible both under acidic and alkaline conditions, though the yield of a target product is usually higher under alkaline conditions.

A p-PPSQ was originally obtained by A. Barry *et al.*^64^ Using basic catalysis, they isolated [PhSiO_1.5_]_*n*_, *n* = 6. However, it was found after clarifications that the product contained 8 rather than 6 units. Later, Brown^65^ isolated and described all the p-PPSQ presented in Fig. 9. Currently, p-PPSQ are usually obtained by basic^66^ or acid hydrolysis^67^ of phenyltrichloro- or trialkoxysilane. T_8_ is the main product of this reaction. To obtain p-PPSQ with other sizes, a more careful selection of the synthesis conditions is required. A synthesis of T_12_ was already described above.^47^ An approach to a synthesis of T_10_ was developed by R. Laine *et al.*^68^ Under optimal conditions, the yield of T_10_ reached about 50% (after isolation). The approach involves the use of tetrabutylammonium fluoride (TBAF) as a catalyst. In that work, the rearrangement of T_8_ in the presence of 1–10% mol. TBAP at various temperatures and in various solvents was studied in detail. Based on the data obtained, three possible mechanisms were assumed. It is better to use the original publication to learn more about them. Here, we only point out that this is a complex process in which the rearrangement involves the breakdown of some polyhedron faces and the formation of new ones. This results in numerous intermediates, as confirmed by MALDI-ToF and ^19^F NMR spectroscopy.

**Fig. 10:**
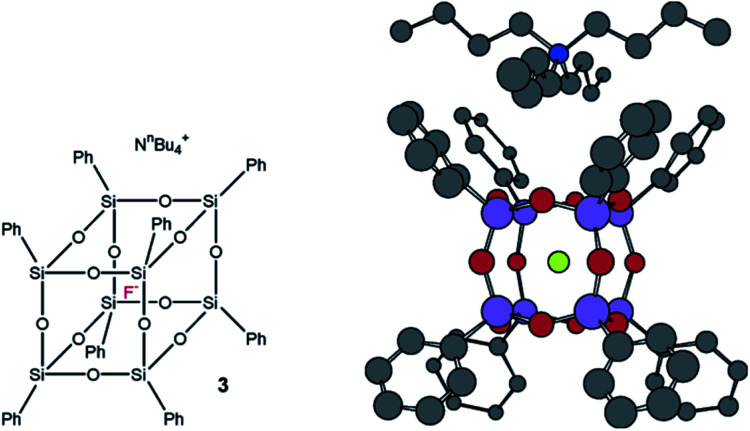
Octaphenyloctasilsesquioxane with a fluoride anion inside and a tetrabutylammonium counterion outside. Reprinted with permission from ref. 70. Copyright 2020 American Chemical Society.

It should be noted that Bassindale^69–71^ was the first to use TBAP for the synthesis of p-PPSQ. The resulting octaphenyloctasilsesquioxane had a very unusual feature. A fluoride anion was arranged inside its molecule (Fig. 10):

The compound thus obtained has a much better solubility due to the presence of the tetrabutylammonium counterion. This fact may expand the scope of p-PPSQ application.

The structure with encapsulated F^−^ is confirmed by single-crystal X-ray crystallography data, as well as ^1^H, ^19^F, ^29^Si NMR and MALDI-ToF data.

**Fig. 11:**
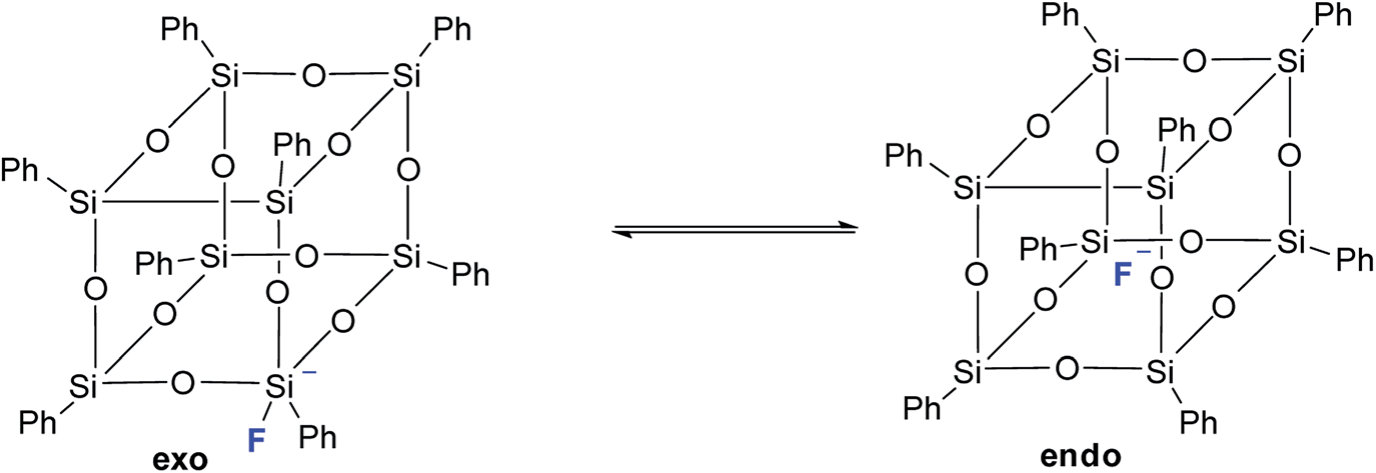
*Exo* and *endo* forms of p-PPSQ with F^−^ (ref. 68).

It should also be noted that in the above work by Laine,^68^ neither T_8_ nor any other cell-like compounds with fluoride encapsulated inside were detected. However, T_8_ with a five-coordinated silicon atom in one of the corners was detected as a transition state. Based on the data obtained, the authors suggested the structure of such a compound with an *exo*-bound fluorine atom, Fig. 11:

As noted above, p-PPSQ alone did not attract much interest of scientists, except for applied researchers for whom the “cubes” turned out to be a kind of “magic dust”, a magic powder that improves the properties of everything: numerous polymer formulations for adhesives, coatings, *etc.*^72–75^ It should be noted here that, whether consciously or not, these compounds are used in such formulations as a molecular nanoscale filler.

The situation changes upon their modification. Incorporation of functional groups into the organic component by electrophilic substitution reactions allows p-PPSQ to be subsequently used for synthesizing a variety of compounds. The most common of these reactions are nitration^76,77^ followed by reduction to an amino group, as well as halogenation.^78–81^ The feasibility of performing this reaction is rather unusual. This is due to the fact that reactions of this kind usually result in the electrophilic substitution of silicon at the *ipso* position of the phenyl ring. However, in the case of p-PPSQ, the preservation of the silsesquioxane core may be due to its high thermodynamic stability, as well as its screening by phenyl substituents. Moreover, the silsesquioxane core exhibits strong acceptor properties comparable in strength to those of the CF_3_ group.^82^ The presence of functional groups at the aromatic core of p-PPSQ makes it possible to subsequently incorporate nearly any organic and inorganic functional and non-functional groups into these compounds. Such transformations significantly expand the scope of p-PPSQ practical use.

**Fig. 12:**
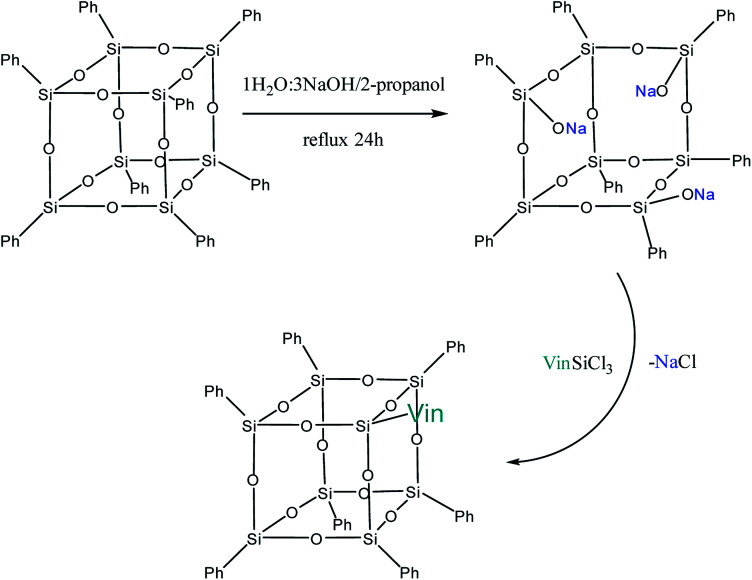
Octaphenyloctasilsesquioxane apex opening reaction.

Yet another modification approach involves a partial destruction of the inorganic core by reactions of the latter with strong bases. Japanese researchers^83^ obtained T_8_ with one open apex by this method. The reaction of such a compound, for example, with VinSiCl_3_, can give a monofunctional p-PPSQ (Fig. 12). Other organic substituents can be incorporated in the same way.^84^ To some extent, this operation makes them related to another class of unique organoelement polycycles, namely, carboranes.^85–88^

**Fig. 13:**
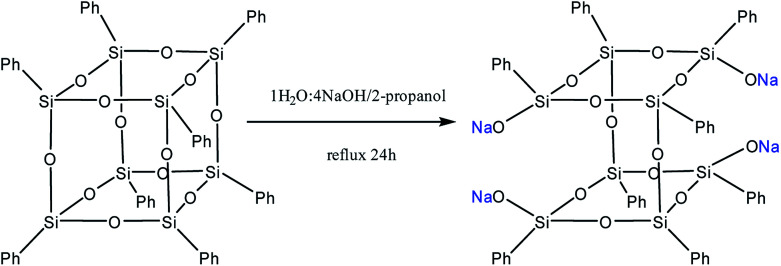
The reaction of opening of two faces in octaphenyloctasilsesquioxane.

**Fig. 14:**
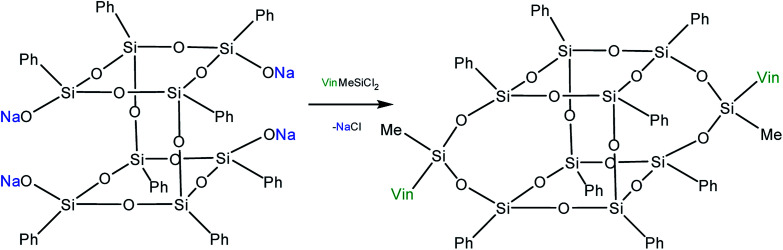
Reaction of sodium octaphenyltetrasiloxanolate with a functional trichlorosilane.

Moreover, two faces can be opened by increasing the amount of the alkali (Fig. 13). Modification of such a compound with various functional organochlorosilanes (Fig. 14) allows one to obtain a difunctional phenylsilsesquioxane monomer. It should be noted that this compound can also be synthesized by alkaline hydrolysis of phenyltrialkoxysilane.^89,90^ The polymers obtained from it have excellent thermal, optical and hydrophobic properties.^91^

Thus, p-PPSQ are yet another form of PPSQ. The polyhedral structure results in a low solubility of these compounds. This differs them from ladder PPSQ and clearly demonstrates the structure–properties relationship. The potential of p-PPSQ is realized upon their modification by electrophilic substitution in the aromatic ring. Another way of modification involves the opening of the silsesquioxane core in one or two places followed by functionalization (Fig. 13 and 14). This allows one to obtain mono- and difunctional compounds. Such functional p-PPSQ are widely used in polymer chemistry and in materials science.^92–101^

### 2.3. Macrocyclic phenylsilsesquioxanes

**Fig. 15:**
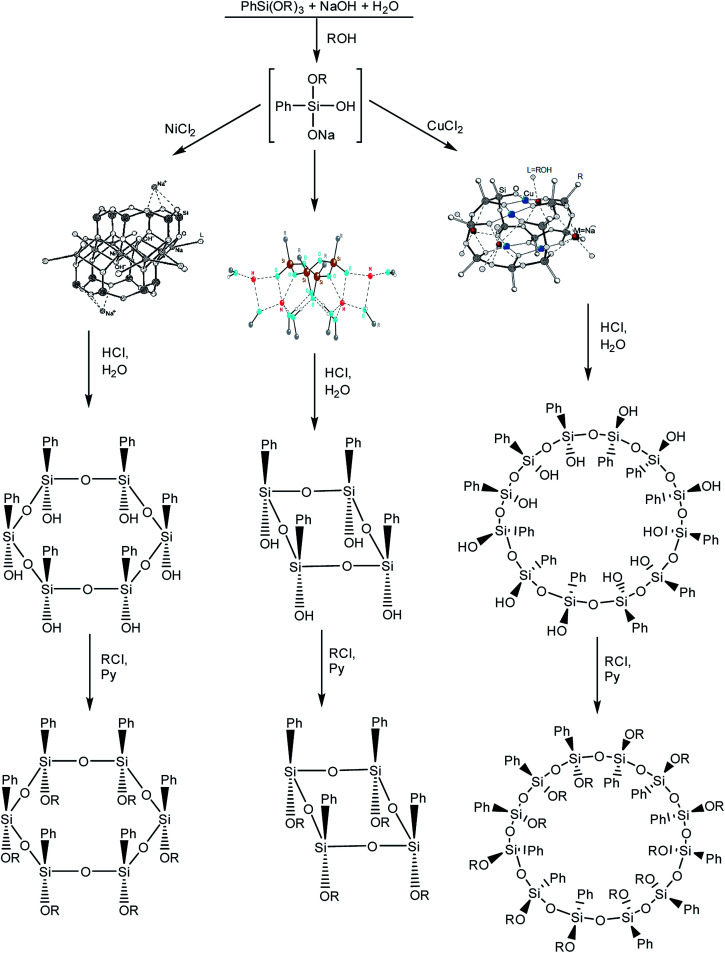
Scheme of the synthesis of macrocyclic phenylsilsesquioxanes with various sizes. Reprinted with permission from ref. 103. Copyright 2020 American Chemical Society.

It is logical to go from polyhedra to siloxane macrocycles that are formed in the syntheses of stereoregular metallosiloxane frameworks.^102^ The synthesis of macrocycles is based on the acid hydrolysis of metallosiloxanes that are, in turn, obtained by alkaline hydrolysis of phenyltrialkoxysilane (Fig. 15 (1–3)). In this case, the addition of various transition metal salts at the second stage of the synthesis determines the size of the future macrocycle. The most general and already classical scheme is given below:^103^

As one can see, further modification of a macrocyclic polyol results in a stereoregular macrocyclic phenylsilsesquioxane.^104^ Functional stereoregular macrocycles are in great demand for the synthesis of numerous derivatives.^105^ In fact, they became a versatile multipurpose platform for synthesizing new polymeric forms, for example, star-shaped polymers^106,107^ and analogs of calixarenes.^108^ Some hydroxy derivatives of macrocycles exhibit the properties of plastic crystals due to a specific organization of hydrogen bonds.^109–113^ This direction deserves a separate discussion that is beyond the scope of this review.

It should be noted that in this case, the template approach was applied much earlier than the idea of templates was used in a synthesis of l-PPSQ.^114^ Essentially, both teams exploited the tendency of hydrolysis products of a trifunctional phenylsilane to form ordered structures in the presence of an additional coordinating agent.

Analysis of the above information leads to the conclusion that by varying the synthesis conditions, namely the temperature, pH of the medium, concentration and ratio of the starting reagents, one can obtain phenylsilsesquioxanes with various structures, *i.e.*, ladder, polyhedral, and macrocyclic ones. In fact, high-temperature polymerization leads to the formation of a predominantly ladder structure, whereas polyhedral compounds are formed at room temperatures and at strong dilution. In this case, three-functional monomers such as phenyltrichlorosilane or phenyltrialkoxysilane are used as the starting components.

### 2.4. PPSQ with complex structural composition

Despite the obvious attractiveness of ladder and polyhedral PPSQ that is due to their highly regular structure, one should make statements about their serious practical prospects with considerable caution. This is primarily due to the complexity and laboriousness of their synthesis. In view of this, there are many studies suggesting variants that are more tempting from a practical point of view.

E. Lesniak *et al.*^115^ obtained polyphenylsilsesquioxanes by hydrolysis of phenyltrichlorosilane under various conditions (random-PPSQ or r-PPSQ).

In all the cases, thermostable amorphous resins (5% weight loss at 410–480 °C) with molecular weights not exceeding 7000 were obtained. Unlike rigid ladder structures, the reported materials had relatively low glass transition temperatures and softening points, namely, 100 and 250 °C, respectively, which makes them promising thermosetting materials due to the ability to undergo curing during heat treatment.

Based on the data of ^1^H, ^29^Si NMR and IR spectroscopy as well as gel permeation chromatography (GPC), it was concluded that all the r-PPSQ obtained had a defective polyhedral or ladder structures. Moreover, all the products contained low molecular weight cyclic components and 1–4% residual Si–OH groups. This implies that all the products obtained contained not only polyhedral and ladder components but also mixed defective structures.

The sol–gel process is yet another method for the preparation of practically interesting polyphenylsilsesquioxanes. Thermoplastic and thermosetting spherical particles were obtained^116,117^ by two-stage acid/base hydrolysis of phenyltriethoxysilane. Based on X-ray diffraction and ^29^Si NMR spectroscopy data, it was concluded that the structure of the polyphenylsilsesquioxane obtained is close to a ladder one. It is difficult to imagine that rod-like ladder structures are capable of forming spherical particles. Probably, this conclusion was based on the absence of silanol groups in the product as well as X-ray scattering data that are consistent with the data of other researchers who obtained ladder l-PPSQ by directed synthesis.

**Fig. 16:**
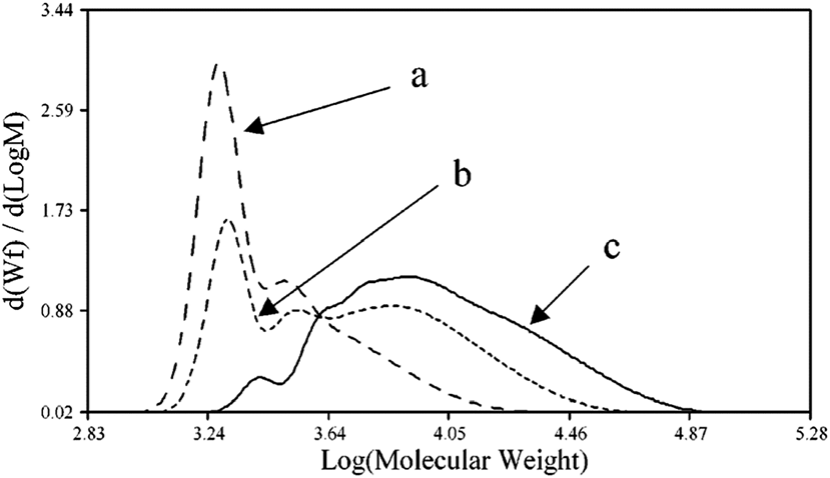
Molecular weight distribution of the main hydrolysis products of phenyltriethoxysilane in the presence of the following catalysts: (a) – Me4NOH, (b) a mixture of Me_4_NOH with octylamine and (c) octylamine. Reprinted with permission from ref. 118. Copyright 2020 American Chemical Society.

John H. Harreld *et al.*^118^ found that by varying the nature of the base and the pH of the medium, one can significantly affect the molecular weight characteristics and structure of phenyltriethoxysilane hydrolysis products. For example, using Me_4_NOH, octylamine or their mixture, one can control the molecular weight of the resulting polymer in a wide range (Fig. 16):

It is noteworthy that T_12_ was the main component of the polymer obtained by method (a). The rest was believed to consist of incompletely condensed polyhedral compounds.

It can be concluded from the above data that the polyphenylsilsesquioxanes obtained by catalytic hydrolysis of the PhSiX_3_ monomer constitute an intermediate case between polyhedral and ladder compounds of this type. In addition, all of the compounds described above have polycyclic structures. The complexity and laboriousness of interpreting the structure and composition of the products should also be mentioned.

It can be seen from the examples considered above that, as a rule, PPSQ form polycyclic structures. One can obtain degenerate structures, for example, ladder or polyhedral ones, by changing the synthesis conditions. Moreover, the intermediate option, *i.e.*, a polymer containing both ladder and polyhedral units, is most attractive for practical application. Their synthesis is much simpler, but the required product properties can only be achieved by empirical tuning of the conditions. Moreover, in contrast to degenerate PPSQ, their structural composition is very complex.

The potential of ladder and polyhedral PPSQ is unlocked upon further modification. The practical value of non-modified PPSQ is still insignificant, while the methods for modifying these PPSQ are rather complicated, ambiguous, and require further development.

## 3. Acyclic form of PPSQ

### 3.1. Methods for producing hyperbranched PPSQ

Hyperbranched polymers are among the most interesting acyclic objects that can be obtained from monomers with more than two functions. As concerns the structural forms of PPSQ, the acyclic form is degenerate, like the ladder and polyhedral ones. In other words, in contrast to the above PPSQ that have structures entirely consisting of cyclic moieties, a hyperbranched a-PPSQ does not contain rings (or contains only one). Therefore, the preparation of hyperbranched a-PPSQ makes it possible to see how changes in structure affect their properties and, in the future, expand the scope of their application.

Hyperbranched polymers increasingly attract the attention of scientists. This interest is due to their unusual architecture, and hence, a combination of unique properties: high solubility, abnormally low viscosity of solutions, low dependence of hydrodynamic radius on molecular weight, high concentration of functional groups on the periphery, and ability to encapsulate guest molecules.^119^ These qualities predetermine the scope of their application as fiber-optic materials, containers for various compounds, catalysts, nanoscale blocks and membranes.^120^ In addition, the possibility of performing polymer-like transformations on peripheral functional groups allows one to give nearly any desired properties to these polymers.

**Fig. 17:**
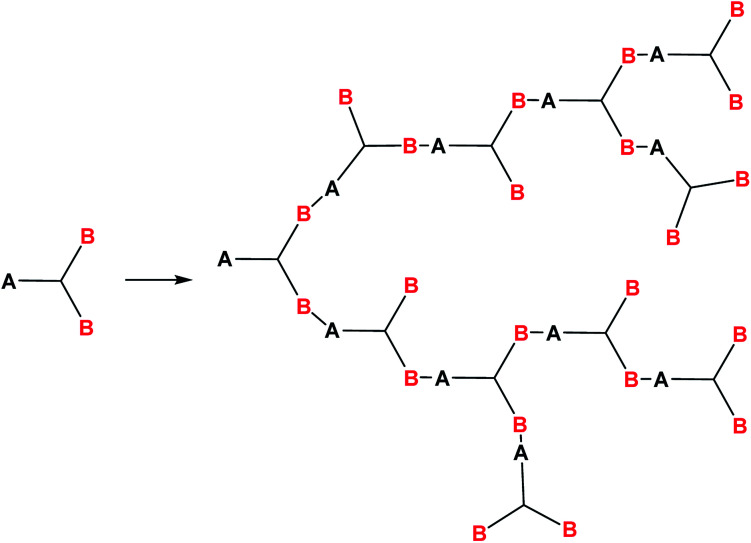
Scheme of the formation of a hyperbranched polymer.

It is known^121^ that polymers of this type are formed in reactions of AB_2_ type monomers where functional group A can react only with functional group B, Fig. 17:

This condition formulated by Flory^121^ allows one to avoid the formation of spatially cross-linked and polycyclic structures.

Despite the prospects of hyperbranched polymers, there are not many examples where hyperbranched polysiloxanes (HBPS) with this structure were synthesized. Some examples where hyperbranched methylsilsesquioxanes were obtained by the Piers−Rubinsztajn reaction are known.^122^

**Fig. 18:**
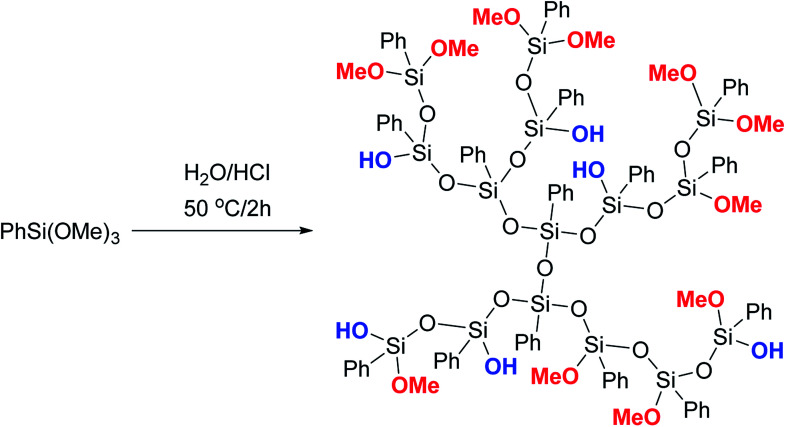
Scheme of synthesizing a phenylsilsesquioxane hyperbranched polymer.^124^ Reproduced with the permission of John Wiley and Sons.

Phenyltrimethoxysilane was subjected to hydrolytic polycondensation under acidic conditions, Fig. 18:^123,124^

It can be assumed, though the article does not provide sufficient evidence of this, that the AB_2_ monomer PhSi(OMe_2_)OH was formed *in situ* in the system and then underwent heterofunctional condensation in the acid medium. It should also be noted that the PTMS/H_2_O/HCl ratio is not reported, though it is likely to play a key role.

Thus, this method cannot be considered satisfactory for building a hyperbranched structure. This work requires a more detailed analysis. Most likely, the PPSQ obtained in this way should be attributed to highly branched rather than hyperbranched systems. At first glance, the difference is small, but it is actually fundamental. The formation of a hyperbranched structure requires compliance with the Flory condition.^121^ It such case, the resulting polymer is acyclic by definition. On the other hand, all systems with a high content of functional groups but at the same time containing an unknown number of cyclic moieties, as well as functionalized linear polymers of the matrix should be attributed to highly branched ones. Unfortunately, the authors neglect this rule. Later, this served as the basis for a whole series of new works based on this method.^125–129^

**Fig. 19:**
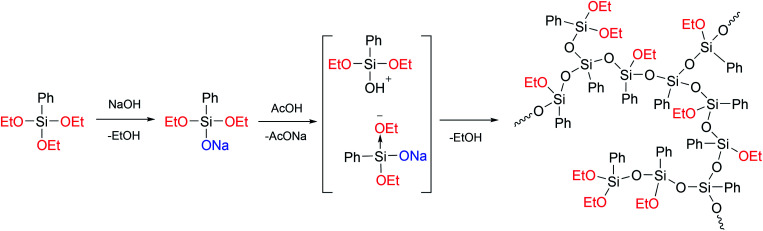
Scheme for the preparation of a hyperbranched polyphenyl(ethoxy)-silsesquioxane.^24^ Reproduced with the permission of Elsevier publishing.

In ref. 24, a previously developed method for the preparation of hyperbranched polysiloxanes^130–132^ was used to obtain a hyperbranched a-PPSQ based on sodium phenyldiethoxysilanolate, Fig. 19:

As one can see from the figure, the AB_2_-type monomer obtained upon neutralization undergoes heterofunctional condensation of the Si–OAlk and Si–OH groups. In this case, the silanol attacks the activated (due to electron density redistribution) alkoxy group available in sodium phenyldialkoxysilanolate that has not been neutralized yet.

**Fig. 20:**
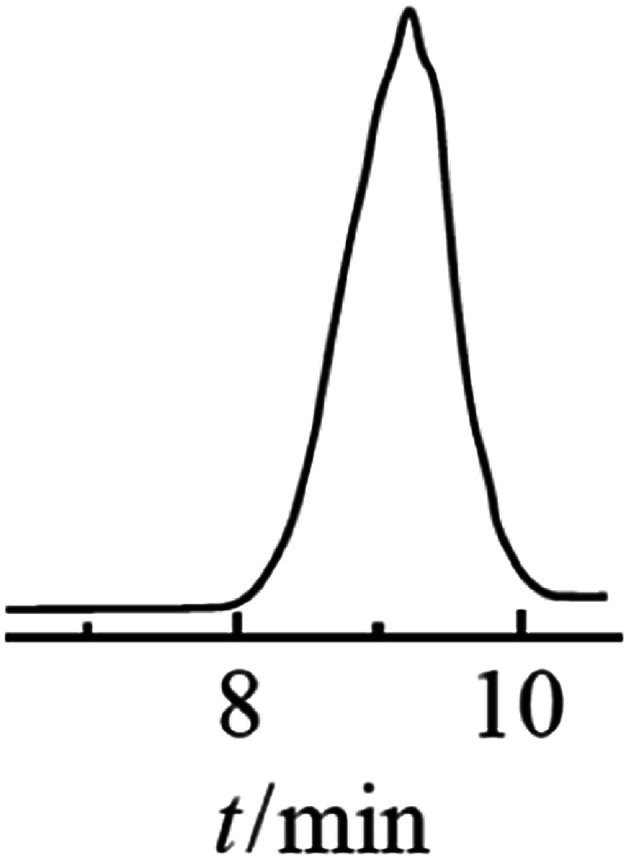
GPC curve of the polyphenyl(ethoxy)siloxane obtained, MW = 1000.^24^ Reproduced with the permission of Elsevier publishing.

**Fig. 21:**
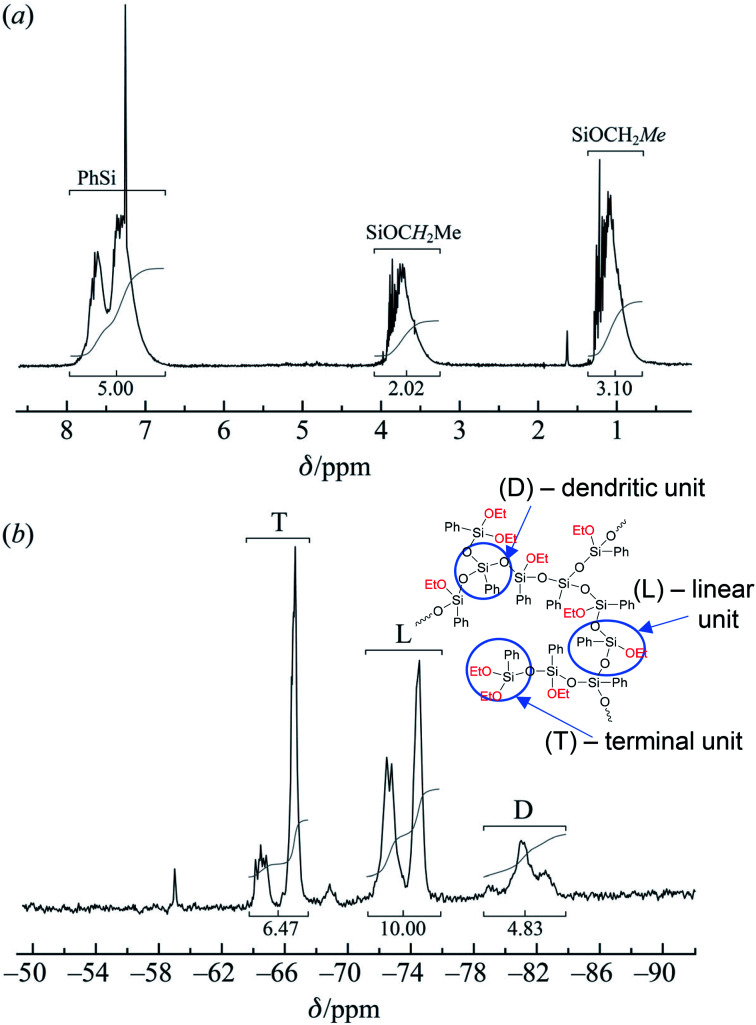
(a) ^1^H and (b) ^29^Si NMR spectra of the polyphenylethoxysiloxanes obtained: D – trisubstituted silicon atom; L – disubstituted silicon atom; T – monosubstituted silicon atom.^24^ Reproduced with the permission of John Wiley and Sons.

The resulting compound was studied by GPC (Fig. 20) as well as ^1^H and ^29^Si NMR spectroscopy (Fig. 21):

As one can see from Fig. 21, the ratio of integral intensities of phenyl and ethoxy group protons in the ^1^H NMR spectrum of the reaction product corresponds to a –[PhSi(OCH_2_CH_3_)]– unit, which confirms that the condensation of the monomer is predominantly heterofunctional and indicates that a hyperbranched polymer is formed. According to the ^29^Si NMR spectrum (Fig. 15 (2)), the resulting compound has three types of silicon atoms, of which the signal corresponding to the silicon atom in the –[PhSi(OCH_2_CH_3_)-O]– unit has the maximum intensity. The signals corresponding to mono- and tri-substituted silicon atoms in the –[PhSi-O]_1.5_– and –[PhSi(OCH_2_CH_3_)_2_-O]– units have similar intensities, which correlates with the data of the ^1^H NMR spectrum. In other words, the structure of the polycondensation product contains dendritic phenylsilsesquioxane units (D), terminal phenyldiethoxysilyl units (T), and linear phenyl(ethoxy)silyl units (L). An important parameter for evaluating hyperbranched polymers is their degree of branching, DB.^133–135^ This characteristic determines parameters such as glass transition temperature, mechanical strength, viscosity of solutions and melts, and ability to encapsulate monomeric compounds. The degree of polymer branching is quantitatively defined as the ratio of the sum of “ideal” dendritic and terminal units to the total number of units in the polymer. The degree of branching equals 1 for a dendrimer structure; for more defective hyperbranched structures, this value usually ranges from 0.35 to 0.7. For the polymers obtained, this value is approximately 0.5. According to GPC data, the resulting product has low molecular weights. This is typical of dense globular systems whose molecular weight characteristics were measured against linear polystyrene standards.

Functional ethoxy groups can be further converted to non-functional triorganosilyl groups by one of the methods (Fig. 23) that will be discussed below in more detail. The figure shows the IR and ^1^H NMR spectra of non-functional hyperbranched phenylsilsesquioxane with terminal Me_2_PhSiO groups.^25^

**Fig. 22:**
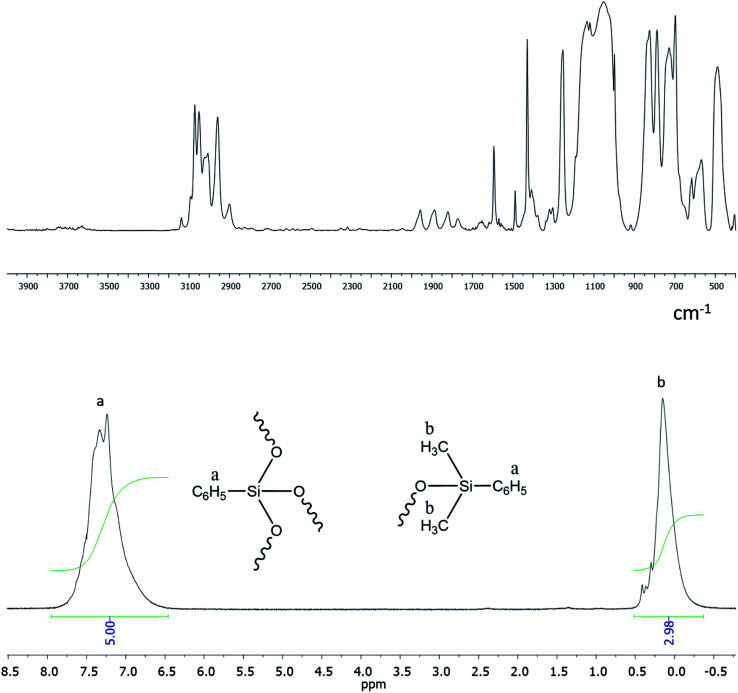
FTIR spectrum of a-PPSQ (top) and ^1^H-NMR spectrum (bottom) of a-PPSQ.^25^ Reproduced with the permission of Elsevier publishing.

It follows from IR spectroscopy data (Fig. 22, top) that the products obtained do no manifest signals corresponding to the absorption band of Si–OH groups in the region of 3300–3800 cm^−1^. This fact qualitatively distinguishes this method of HBPS synthesis from the previous work^124^ where a-PPSQ were obtained by acid hydrolysis of phenyltrimethoxysilane. Obviously, in the case of acid hydrolysis, the final product is likely to have many residual silanol group or cyclic moieties.

According to ^1^H NMR data (Fig. 22, bottom), one can see that the ratio of PhSi and MeSi groups does not change and remains equal to 1 : 1.

Thus, it can be seen that the method of synthesizing a-PPSQ from sodium phenyldiethoxysilanolate has an advantage over other approaches. In this case, we use an AB_2_ monomer that satisfies the Flory condition. This results in the formation of solely hyperbranched polymers.

Moreover, this approach is rather simple and versatile. It can be used to obtain both a-PPSQ and a number of other hyperbranched organosilsesquioxanes.^136–138^

### 3.2. Modification of a-PPSQ

As shown above for ladder and polyhedral PPSQ, their potential can be significantly increased by modification. a-PPSQ is not an exception in this respect.

The a-PPSQ obtained can be further modified both at the peripheral groups and by means of intramolecular condensation.

**Fig. 23:**
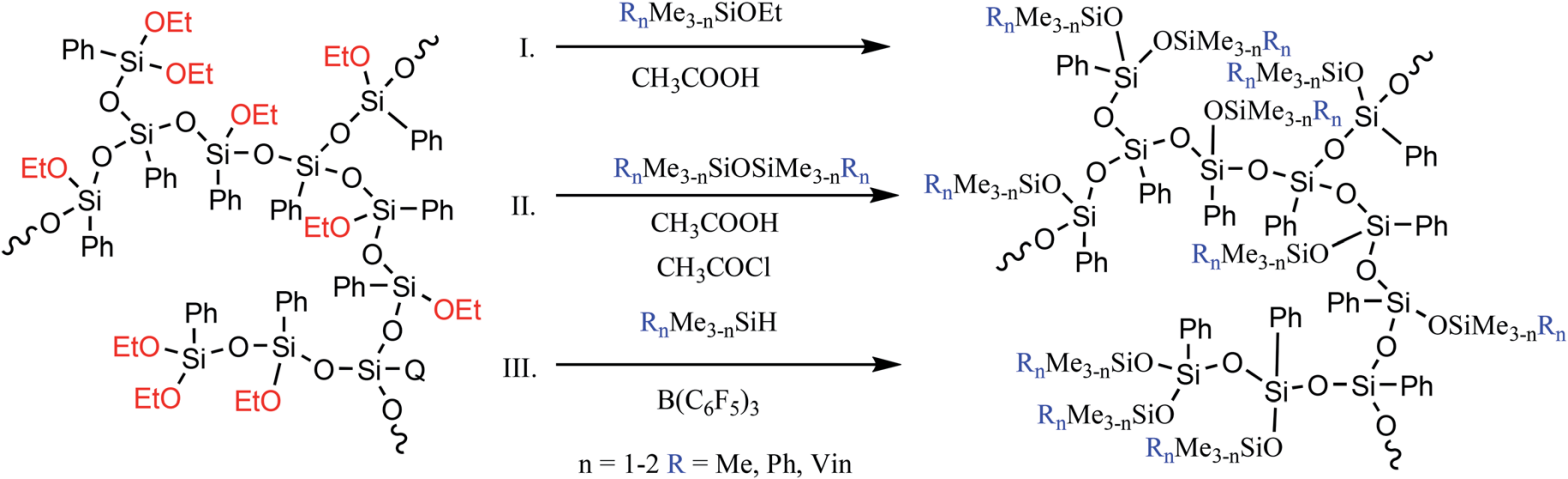
Schemes of variants for synthesizing a non-functional modification of the polymer obtained.

The former approach results in triorganosiloxy-substituted a-PPSQ. In this case, there are no special restrictions on the type of the triorganosilyl group, *i.e.*, like in the case of polyhedral phenylsilsesquioxanes with a functionalized aromatic core, it then becomes possible to incorporate nearly any other group. Such nonfunctional derivatives can be obtained by conducting the reaction in an active medium using a monofunctional alkoxysilane (Fig. 23 (I)) or a symmetric triorganodisiloxane (Fig. 23 (II)).

The active medium is understood as acetic acid that acts simultaneously as a solvent, a reagent, and a catalyst. Total conversion of alkoxy groups is achieved, regardless of their nature.^139^ The first two methods have advantages and drawbacks. The advantage of the former is that no additional chlorine-containing catalyst is used. At the same time, a much cheaper disiloxane is used in the latter method. The drawbacks of methods I and II include the need for a large excess of a monofunctional reagent. Moreover, even a large excess does not exclude the possibility of intramolecular cyclization. At the same time, their overall advantage lies in the ability to finely control the structure of the final product. Depending on the amount of the blocking agent used in the reaction and on the time of its addition to the reaction, it is possible either to preserve the hyperbranched structure of the initial matrix or to transform it into a nanogel structure with various densities of the polycyclic core. The latter circumstance is very important since it determines the set of properties of the final product, such as the glass transition temperature and the activation energy of viscous flow.^137,140^ This issue will be discussed in more detail below.

The third method (Fig. 23 (III)) guarantees the production of a completely acyclic polymer and involves the Piers−Rubinsztajn reaction between Si–OAlk and Si–H groups.

It was demonstrated^24^ that the choice of the terminal trimethylsiloxy group had a significant effect on the glass transition temperature and on the thermal and thermooxidative stability of the final product (Table 1):

**Table 1:** Thermal properties of a-PPSQ with various terminal groups

Terminal group	MW	*T*_glass_, °C	*T*_5% mass loss_, °C
Me_3_SiO–	1100	−58.00	220
VinMe_2_O–	1000	−49.00	250
MePh_2_O–	1000	2.50	375

Table 1 shows that on transition from trimethylsilyl groups to methylphenylsilyl ones, *T*_c_ increases by 60 °C and the 5% mass loss temperature increases by 135 °C.

This suggests that it is possible to control the properties of hyperbranched a-PPSQ in a wide range. Moreover, the selection of various organic groups can be very convenient for preparing new formulations with a-PPSQ.

**Fig. 24:**
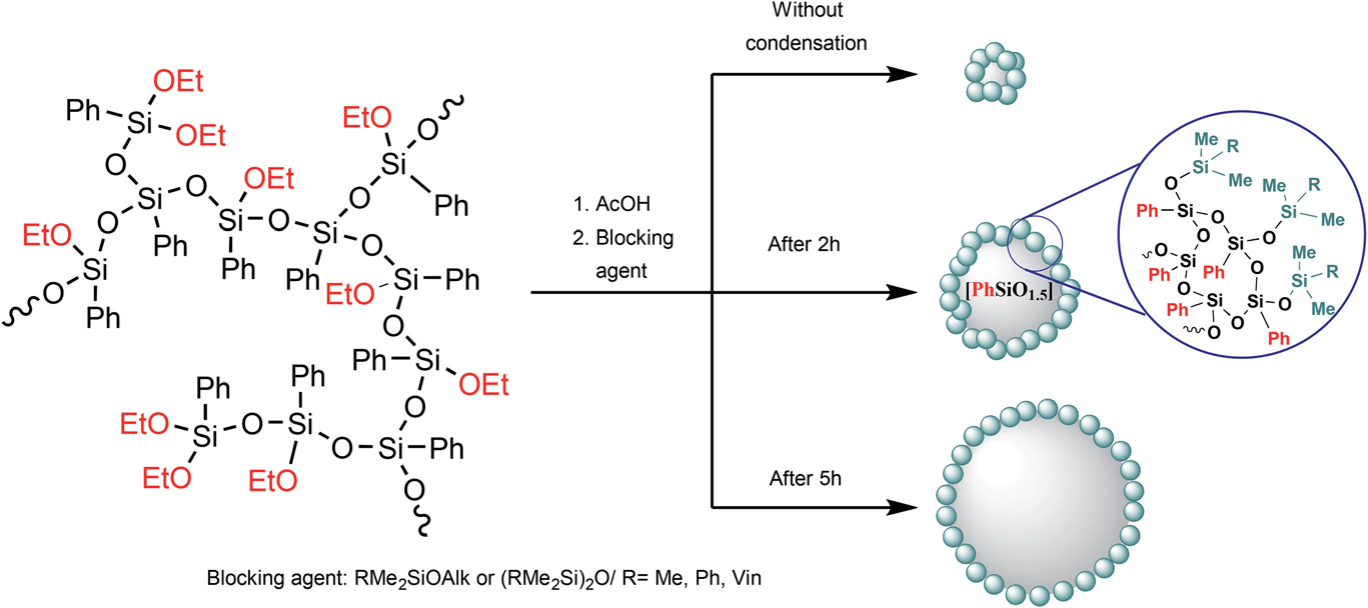
Production of n-PPSQ with various core/shell ratios.^24^ Reproduced with the permission of Elsevier publishing.

In the case of intramolecular condensation, so-called nanogels with a densely crosslinked phenylsilsesquioxane core (nanogel-PPSQ or n-PPSQ) and a triorganosilyl shell are formed (Fig. 24):

This occurs due to the hydrolytic polycondensation of alkoxy groups.^24,130,139^ It occurs both in intra- and intermolecular manner. At a certain stage, reflux is stopped and a monofunctional (blocking) reagent is added. The duration of condensation in acetic acid determines the molecular weight of the product. The compounds obtained in this way are core/shell systems where the core contains an organosilsesquioxane (RSiO_1.5_) unit, while the shell contains a triorganosiloxy unit (R_3_SiO_0.5_).^130^

**Fig. 25:**
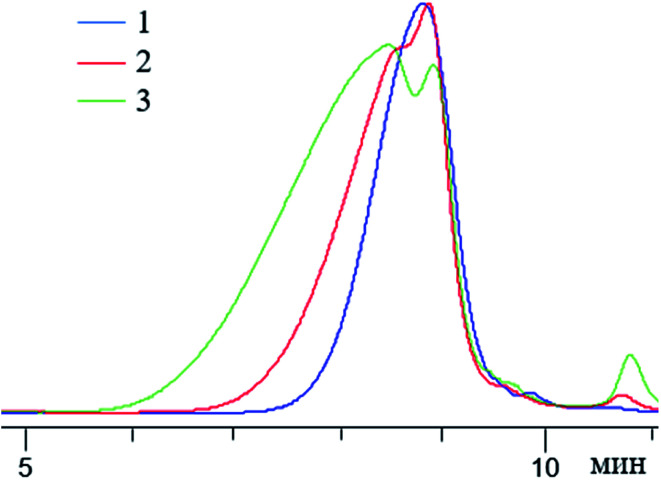
GPC curves of n-PPSQ blocked with dimethylvinylsilyl groups without preliminary condensation ((1) MW-1000), after 2 hours ((2) MW from 10 000 to 1200) and after 5 hours of condensation ((3) MW from 32 000 to 1500).^24^ Reproduced with the permission of Elsevier publishing.

Fig. 25 shows how the condensation time affects the molecular weight of nanogels.

One can see that with an increase in the condensation time, the MW of the product increases and the MWD broadens.

**Fig. 26:**
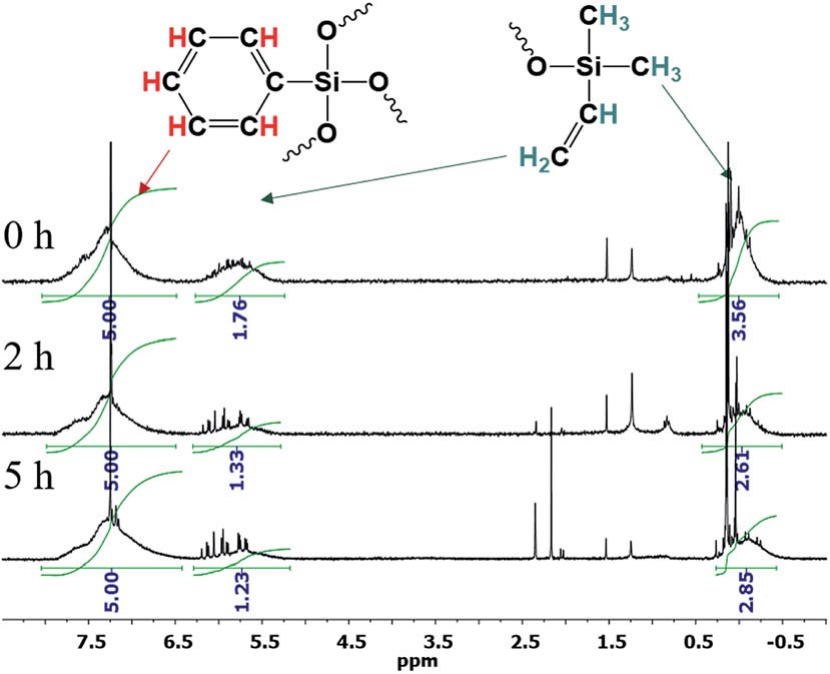
^1^H NMR spectra of n-PPSQ blocked with dimethylvinylsilyl groups without preliminary condensation (1), as well as after condensation for 2 h (2) and 5 h (3).^24^ Reproduced with the permission of Elsevier publishing.

The ^1^H NMR spectra show that the core/shell ratio changes, Fig. 26:

One can see that the core/shell ratio increases with the duration of condensation in the active medium. The integral intensity of protons belonging to the phenyl group of the core increases compared to the protons of the vinyl group located at the molecule periphery. This may indicate that the crosslinking density of the nanogel core increases and, at the same time, intermolecular condensation occurs in parallel with intramolecular cyclization.

It is important to note that the structure of these nanogels is unambiguous. They consist of nanometer-sized globular objects. Compounds of this type can only be obtained from a-PPSQ. This is because the condensation of a monomer, PhSi(OAlk)_3_ for example, will give a set of structural units. As shown above, these are ladder and polyhedral moieties along with intermediate units (*i.e.*, hydroxyl-containing, incompletely condensed polyhedra, and defective ladders).

Modification of peripheral functional groups largely determines the further field of application of the resulting n-PPSQ. For example, if the surface layer of the phenylsilsesquioxane nanogel is blocked with dimethylvinylsilyl units, it becomes possible to use it in further transformations by hydrosilylation.

**Fig. 27:**
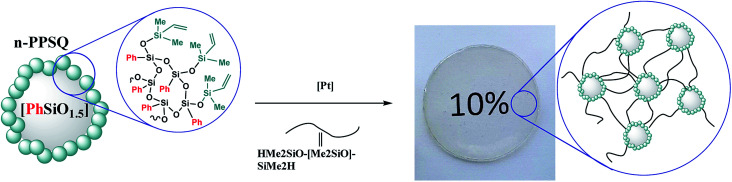
Scheme of PDMS and n-PPSQ phenylated copolymer curing.^141^ Reproduced with the permission of Elsevier publishing.

For example, a nanogel with a dimethylvinylsilyl shell was obtained^141^ for curing polydimethylsiloxane with terminal hydride-containing groups (Fig. 27).

**Fig. 28:**
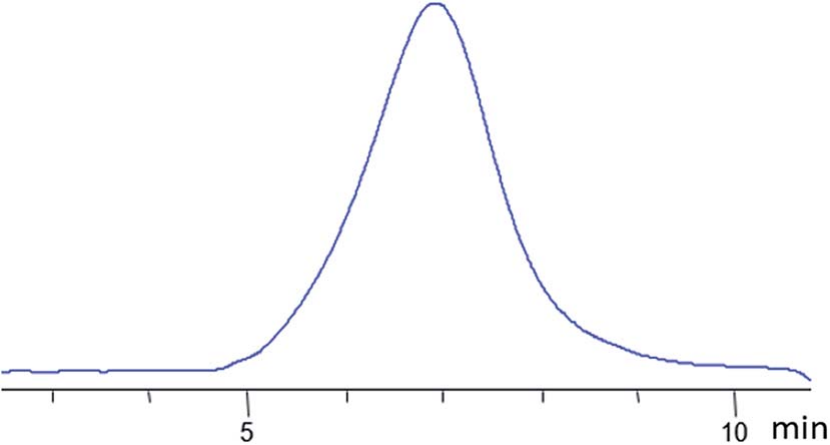
GPC curve of the nanogel with dimethylvinylsilyl shell and MW 8000 obtained.^141^

**Fig. 29:**
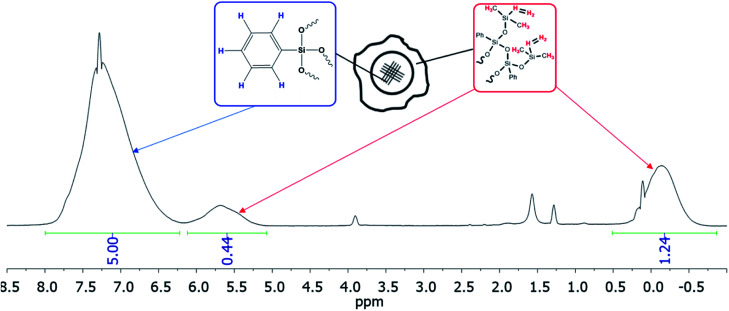
^1^H NMR of the nanogel with a dimethylvinylsilyl shell obtained.^141^

**Fig. 30:**
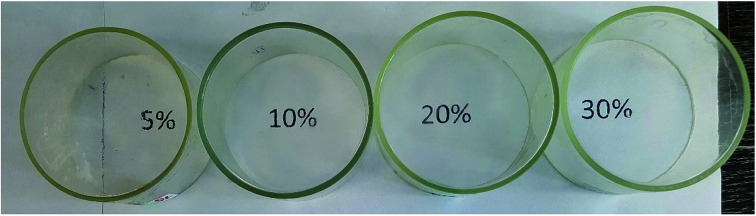
Photographs of films containing 5, 10, 20, and 30% curing agent, respectively.^141^

It was expected that the vulcanizate would have enhanced heat resistance and strength. The nanogel was synthesized by Scheme II (see Fig. 23). The product was isolated by reprecipitation and characterized by GPC (Fig. 28) and ^1^H NMR (Fig. 29):

According to GPC data, the resulting polymer has MW = 8000. One can see from the ^1^H NMR spectrum that the core size is much larger than the shell size. The PDMS used had a molecular weight of 9000. A complex of zero-valent platinum with divinyltetramethyldisiloxane known as the Karsted catalyst was used as the curing catalyst. A solution of the mixture of components along with the catalyst was poured onto a cellophane substrate and hardened for one day. In this way, films with a polyphenylsilsesquioxane nanogel content of 5, 10, 20, and 30% of the PDMS mass, respectively, were obtained (Fig. 30):

Fig. 30 shows that the films formed on the upper part of the rings are completely transparent, as follows from the fact that the inscriptions corresponding to the nanogel content in PDMS that are clearly visible through them.

All the films had a high heat resistance up to 395 °C (Table 2). The amount of the solid residue increases in proportion to the nanogel content from 21% to 57%, respectively (Table 2).

**Table 2:** Properties of films obtained from PDMS and -PPSQ nanogel

No.	Nanogel content in the film (relative to PDMS mass), %	Young's modulus, MPa	Deformation until rupture, %	Temperature of 5% mass loss, °C	Fraction of the solid residue, %
1	PDMS^b^	—a	—a	375	15
2	5	0.06	280	380	21
3	10	0.21	355	375	30
4	20	0.65	380	395	36
5	30	3.6	80	365	57

a Not measured.

b Uncured PDMS.

**Fig. 31:**
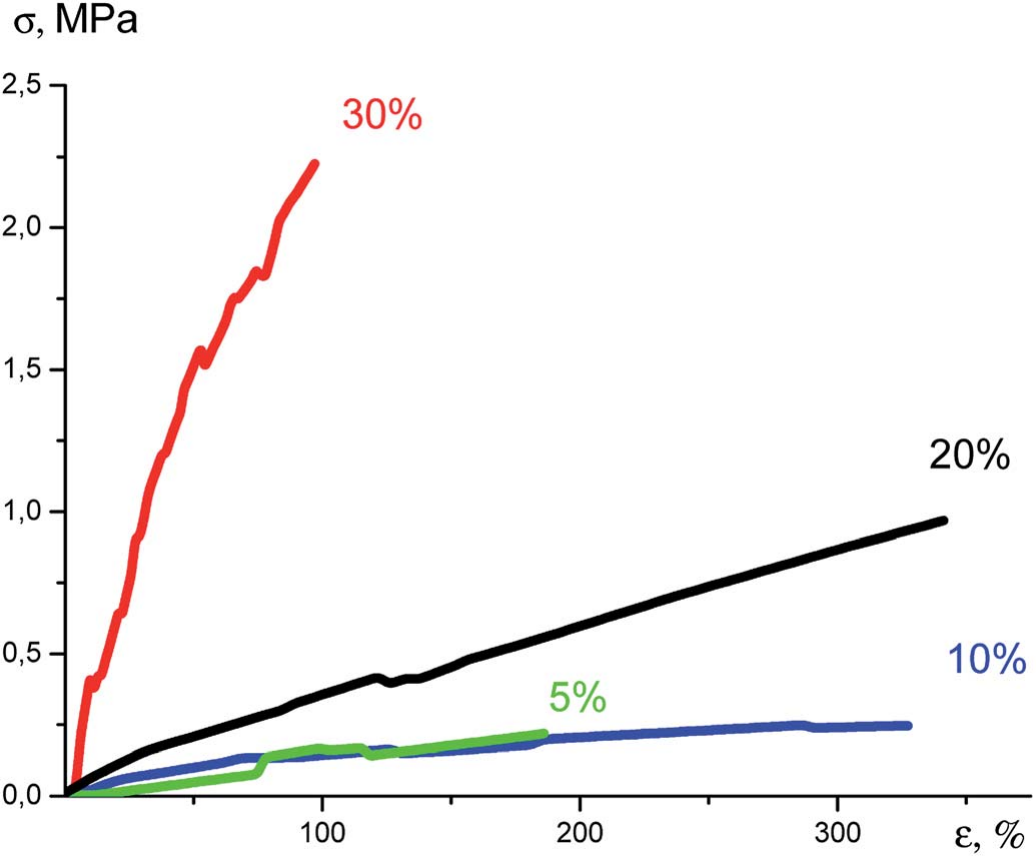
Plots of tensile stress *versus* strain.^141^

An increase in the content of the nanogel in the formulation resulted in significant changes in the Young modulus. It can be seen from the tensile curves (Fig. 31) that on passage from 5% to 30% of the nanogel content, this indicator increases 60-fold. In this case, the deformation until rupture decreases from 380 to 80%, Table 2.

The noticeable changes in the properties of vulcanizates can be explained by the fact that the nanogels that are added serve not only as a cross-linking agent but also as a molecular filler with stronger intermolecular interactions inherent in phenylated systems in comparison to the classical vulcanized PDMS.^142^

Because of the importance of organizing the structure of molecular forms of polyphenylsilsesquioxanes, the structure–property relationship was demonstrated in the coordinates: acyclic polymer–polycyclic polymer and polycyclic polymer–colloidal particle.^25^

**Fig. 32:**
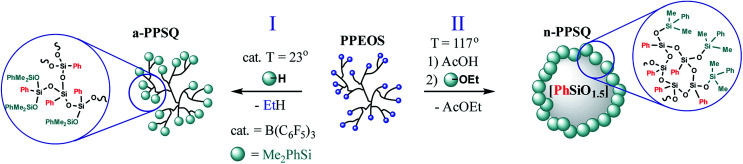
Synthesis of acyclic (I) and polycyclic (II) PPSQ forms.^25^ Reproduced with the permission of Elsevier publishing.

The Peirce–Rubinstein reaction (Fig. 23, Scheme III) was used to obtain a hyperbranched polymer (a-PPSQ), while a reaction in the active medium was used to obtain a polycyclic n-PPSQ, Fig. 32:

The IR and ^1^H NMR spectra of a-PPSQ are shown in Fig. 22. The characteristics of the polymers obtained are shown in Table 3.

**Table 3:** Properties of the PPSQ obtained

No.	Polymer type	MW	*E*_a_, kJ mol^−1^	*η*, at *T* = 150 °C, Pa s	*T*_5% wt loss in air_, °C/solid residue, %	*T*_5% wt loss in argon_, °C/solid residue, %	*T*_g_, °C
1	a-PPSQ	2150	36^a^	0.38	435/40	430/35	−34
2	n-PPSQ	1400	92^a^	1.8	405/41	—b	29
3	n-PPSQ	2860	186	1.5 × 10^4^	441/45	—b	98
4	n-PPSQ	3850	195	2.9 × 10^4^	444/47	490/70	104
5	n-PPSQ	5050	—b	—b	480/47	502/75	148

a The dependence of viscosity on temperature is non-linear in Arrhenius coordinates. The value of *E*_a_ was calculated for the 1/*T* range of 0.0023–0.0028 where the plot is linear.

b Not measured.

**Fig. 33:**
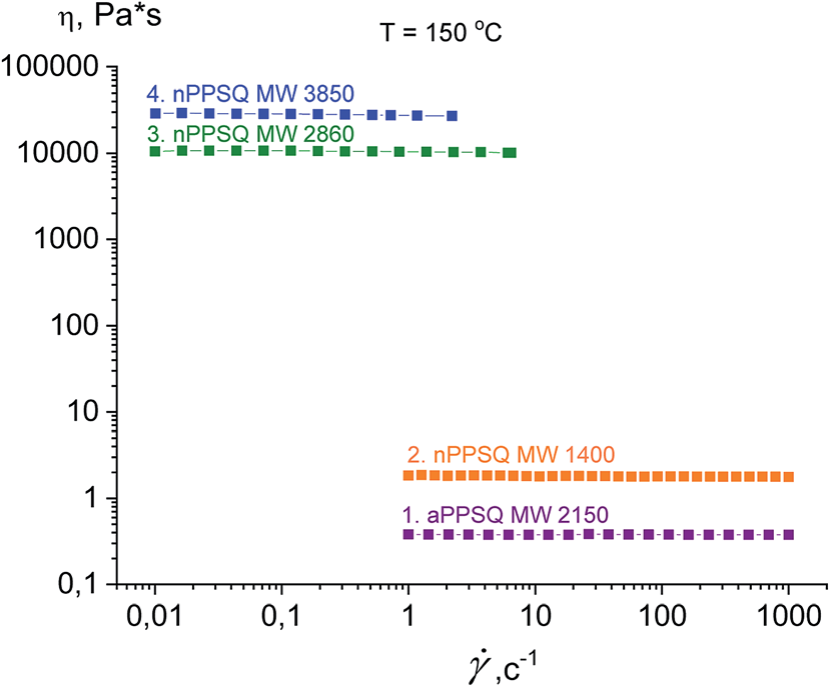
Dependence of viscosity on the shear rate (flow curves) at 150 °C for the PPSQ obtained.^25^ Reproduced with the permission of Elsevier publishing.

The authors draw two conclusions from the data on the dependence of viscosity on the shear rate for the samples obtained (Fig. 33).

The first conclusion is that on transition from MW 1400 to 2860, the dynamic viscosity increases greatly from 1.8 Pa s to 1.05 × 10^4^ Pa s, respectively (Table 3). Such an abrupt change in the physical properties due to minor changes in the chemical composition is a characteristic feature of these objects.^143^ An explanation for this is that as the fraction of the polycyclic core increases, the reptational (segmental) flow turns to colloidal flow where the particle moves as a whole. This phenomenon was previously observed for n-PMSQ,^137,140^ but it is also fully observed for the n-PPSQ obtained.

The second conclusion is that the viscosity of a-PPSQ with MW 2150 kDa is smaller (0.38 Pa s) than that of n-PPSQ with MW = 1400 (1.8 Pa s) (Table 3). The decrease in viscosity is related to the greater segmental mobility of a-PPSQ due to the possibility of rotation around the SiOSi bonds that occurs in acyclic structures and is impossible in polycyclic ones.

One can see from TGA data (Table 3) that all the samples have high thermooxidative stability and that the 5% losses occur in the range of 405–480 °C. In other words, all the PPSQ obtained have high thermal stability, regardless of their structure.

At the same time, the glass transition temperature of a-PPSQ (−34 °C) is significantly lower than those of all n-PPSQ (see Table 3). Like in the case of viscosity, this is due to the significantly higher segmental mobility of structural units in a hyperbranched polymer in comparison with the rigid inactive polycyclic structure of nanogels. This is important because, unlike a-PPSQ, the classical PPSQ have a high *T*_g_ along with high thermal resistance. Accordingly, a hyperbranched structure significantly expands the PPSQ scope of use, in particular, as working fluids and modifiers of liquid formulations. Hence, studies in this field are promising.

Comparison of ladder and nanogel analogs is especially interesting. In this case, the shell is of great importance. The glass transition temperature increases as the core grows. Thus, if this tendency is extrapolated to a zero layer, we will probably come to the conclusion that linear (ladder) and polycyclic (nano-gel) structures show a similar result, *i.e.*, the *T*_g_ is higher than the decomposition temperature.

Thus, like for n-PMSQ with a similar structure, comparison of acyclic and polycyclic globular PPSQ forms shows that a transition from the molecular flow model to the colloid flow model occurs.^137,140^

Although comparison of the new forms with ladder PPSQ is not quite objective since we compared a homopolymer with core–shell copolymers, this is still the best approximation available at the moment. On the other hand, comparison with n-PMSQ is totally objective and allows one to conclude that the molecular structure organization provides similar trends in their changes. A transition from a macromolecule to a particle is evidently manifested in both cases. The effect of phenyl substituents at silicon atoms was also predictable. The incorporation of these substituents increased the thermal stability of polymers with similar structures.

Thus, the new PPSQ forms differ significantly from the reference objects and significantly expand the scope of promising practical applications. For example, they can be used to modify liquid siloxane formulations, both in terms of expanding the temperature range (a-PPSQ) and controlling the viscosity of such formulations (n-PPSQ). The difference between a-PPSQ and n-PPSQ with various molecular weights was demonstrated.

Thus, the possibility of modifying a-PPSQ both by substitution of peripheral functional groups and by intramolecular cyclization of the core with a simultaneous increase in the molecular weight of the macromolecule due to intermolecular condensation was demonstrated. That is, depending on the heterofunctional condensation conditions, one can obtain both hyperbranched functional and non-functional polyphenylsilsesquioxanes and similar nanogels with various crosslinking densities and sizes of the phenylsilsesquioxane core. The properties of the products thus obtained vary greatly depending on the structure.

The glass transition temperature and thermal stability of a-PPSQ vary strongly on passage from one type of blocking groups, Me_3_SiO–, to another type, MePh_2_SiO–. The glass transition temperature and viscosity change noticeably depending on the organization of the core on passage from hyperbranched a-PPSQ to n-PPSQ with a polycyclic core. The viscosity of hyperbranched a-PPSQ is noticeably lower than that of its polycyclic analogs. In this case, an increase in the MW of the nanogel changes the nature of its flow as it is converted into a colloid.

Modification of the shell with dimethylvinylsilyl groups allows one to perform further modifications at the double bond. This was demonstrated for nanogels as an example. The PDMS-based films thus obtained have enhanced strength. A material with a high refractive index based on a nanogel and a copolymer with a complex composition is of interest for optoelectronics.

## 4. Conclusions and prospects

The paper describes the traditional polycyclic forms of PPSQ and compares them with the new acyclic form.

It is shown that the new hyperbranched a-PPSQ has significant potential, both in terms of fundamental and applied science. These benefits are as follows:

(1) The new form is also degenerate as is the ladder form. Unlike l-PPSQ where the structure is entirely polycyclic, a-PPSQ does not contain cyclic units. This results in sharp differences between them that are reflected in significant differences in the glass transition temperatures and mechanical properties.

(2) The synthesis of a-PPSQ is much simpler, which makes it more accessible and puts them on a par with irregular cyclolinear r-PPSQ based on this indicator.

(3) Moreover, in the case of irregular r-PPSQ, the final product properties are controlled purely empirically, while in the case of hyperbranched a-PPSQ and its further transition to the nanogel form, the properties are set by a conscious choice of the synthesis conditions (time of condensation in acetic acid, choice of terminal groups, *etc.*).

(4) The new form of a-PPSQ is versatile because both polycyclic and hyperbranched derivatives with various MWs and chemical framing can be obtained on its basis. Nonfunctional derivatives are promising as plasticizers, components of lubricating formulations, heat carriers, molecular fillers, whereas the use of functional derivatives entirely depends on the type of the blocking agent and has practically no restrictions. The review demonstrates only the options for their use in siloxane formulations, but this is a small part of their potential applications. Taking into account the numerous functional organosilicon modifiers that are applicable within the suggested synthetic approaches, it is possible to program not only siloxane but also new silicon-epoxy, siloxane-urethane, as well as organo-inorganic formulations and materials.

To sum up the general results, we can say that none of the existing forms of PPSQ had such a huge synthetic potential in terms of the variety of functional and nonfunctional derivatives, except for polyhedral metallosiloxanes and stereoregular cycles based on them. However, in terms of the practical application potential and the availability of various forms, globular PPSQ are out-of-competition.

## Conflicts of interest

The authors declare no conflict of interest.

## Supplementary Material
